# Same-Day Tools, Including Xpert Ultra and IRISA-TB, for Rapid Diagnosis of Pleural Tuberculosis: a Prospective Observational Study

**DOI:** 10.1128/JCM.00614-19

**Published:** 2019-08-26

**Authors:** Richard Meldau, Philippa Randall, Anil Pooran, Jason Limberis, Edson Makambwa, Muhammed Dhansay, Aliasgar Esmail, Keertan Dheda

**Affiliations:** a Centre for Lung Infection and Immunity, Division of Pulmonology, Department of Medicine and UCT Lung Institute & South African MRC/UCT Centre for the Study of Antimicrobial Resistance, University of Cape Town, Cape Town, South Africa; b Faculty of Infectious and Tropical Diseases, Department of Infection Biology, London School of Hygiene and Tropical Medicine, London, United Kingdom; University of North Carolina School of Medicine

**Keywords:** IRISA-TB, *Mycobacterium tuberculosis*, ULTRA, Xpert MTB/RIF, adenosine deaminase, interferon gamma

## Abstract

The diagnosis of pleural tuberculosis (TB) is problematic. The comparative performance of newer same-day tools for pleural TB, including Xpert MTB/RIF Ultra (ULTRA), has hitherto not been comprehensively studied. Adenosine deaminase (ADA), IRISA-TB (interferon gamma ultrasensitive rapid immunosuspension assay), Xpert MTB/RIF, and ULTRA performance outcomes were evaluated in pleural fluid samples from 149 patients with suspected pleural TB. The reference standard was culture positivity (fluid, biopsy specimen, or sputum) and/or pleural biopsy histopathology (termed definite TB). Those designated as having non-TB were negative by microbiological testing and were not initiated on anti-TB treatment. To determine the effect of sample concentration, 65 samples underwent pelleting by centrifugation, followed by conventional Xpert MTB/RIF and ULTRA. Of the 149 patients, 49 had definite TB, 16 had probable TB (not definite but treated for TB), and 84 had non-TB. ULTRA sensitivity and specificity (95% confidence intervals [CI]) were similar to those of Xpert MTB/RIF [sensitivity, 37.5% (25.3 to 51.2) versus 28.6% (15.9 to 41.2), respectively; specificity, 98.8% (96.5 to 100) versus 98.8% (96.5 to 100), respectively]. Centrifugation did not significantly improve ULTRA sensitivity (29.5% versus 31.3%, respectively). Adenosine deaminase and IRISA-TB sensitivity were 84.4% (73.9 to 95.0) and 89.8% (81.3 to 98.3), respectively. However, IRISA-TB demonstrated significantly better specificity (96.4% versus 87.5% [*P* = 0.034]), positive predictive value (93.6% versus 80.9 [*P* = 0.028]), and positive likelihood ratio (25.1 versus 6.8 [*P* = 0.032]) than ADA. In summary, Xpert ULTRA has poor sensitivity for the diagnosis of pleural TB. Alternative assays (ADA and IRISA-TB) are significantly more sensitive, with IRISA-TB demonstrating a higher specificity and rule-in value than ADA in this high-TB-burden setting where HIV is endemic.

## Abstract (precis)

The diagnosis of pleural tuberculosis (TB) is problematic. The comparative performance of newer same-day tools for pleural TB, including Xpert MTB/RIF Ultra (ULTRA), has hitherto not been comprehensively studied. Adenosine deaminase (ADA), IRISA-TB (interferon gamma ultrasensitive rapid immunosuspension assay), Xpert MTB/RIF, and ULTRA performance outcomes were evaluated in pleural fluid samples from 149 patients with suspected pleural TB.

## INTRODUCTION

Tuberculosis (TB) remains a global health problem, with 10 million new cases attributable to the disease in 2017 (1). Although pulmonary TB is the predominant form of the disease, extrapulmonary TB (EPTB) accounts for approximately 25% of active cases (2), with pleural TB being a common manifestation of EPTB (3), if not the most common in several settings (4–6). The diagnosis of pleural TB is difficult due to the paucibacillary nature of the disease and the need for invasive sampling, including blind, image-guided, or surgical open pleural biopsy (7). Diagnosis using pleural fluid is, in reality, the norm despite several drawbacks, including limited sensitivity and specificity.

Xpert MTB/RIF, a fully automated quantitative real-time PCR assay that was, until recently, the frontline test for TB in many countries where the disease is endemic (8), had a poor yield in pleural TB (using pleural fluid), with a pooled sensitivity of ∼25% when using culture and pleural biopsy as a reference standard (9–11).

However, more recently Cepheid developed the next-generation Xpert MTB/RIF Ultra (ULTRA), a multiplex nested PCR assay, which is endorsed by the WHO as the new sputum-based frontline TB diagnostic test (12). Its key advantage is a higher sensitivity, with the level of detection decreasing from ∼130 to ∼20 organisms/ml of sample. This ∼log difference in sensitivity provided hope that paucibaciliary TB, including forms of EPTB like pleural TB, could be more easily diagnosed (13). However, there are no comprehensive studies on the utility of ULTRA in pleural TB and none from countries where TB is endemic. A preliminary laboratory-based study detected 10 ULTRA positives (sensitivity of ∼47%) in selected culture-positive pleural fluid samples; given that culture sensitivity is only ∼40%, the key drawback was one of selection and sampling bias (14). Thus, it is unknown how the performance of ULTRA compares with that of the conventional Xpert MTB/RIF assay for the diagnosis of pleural TB in unselected patients.

Given the drawbacks of microbiological tests, biomarkers to aid in pleural TB diagnosis, such as adenosine deaminase (ADA), have been extensively studied (7), although specificity may be limited at the 30-IU/liter cut point (often used in clinical practice) (15). An alternative biomarker, interferon gamma (IFN-γ), an inflammatory cytokine secreted by macrophages and CD4^+^ T cells, becomes highly compartmentalized in TB, with pooled sensitivity and specificity estimates of 93% and 96%, respectively (3), and even higher sensitivities in high-TB-burden settings (16, 17). The IRISA-TB (interferon gamma ultrasensitive rapid immunosuspension assay) is a recently validated and standardized same-day (1.5-h turnaround time), low-cost immunoassay assay developed to measure unstimulated IFN-γ in EPTB. Its performance relative to that of ULTRA had not been evaluated before now.

To address these gaps in our knowledge, we performed unbiased evaluation of the 4th-generation Xpert cartridge (Xpert MTB/RIF), Xpert ULTRA, ADA, and IRISA-TB in consecutively recruited patients in a prospective observational study using a comprehensive composite reference standard comprised of culture and pleural biopsy histology.

## MATERIALS AND METHODS

### Patient recruitment, categorization, and routine laboratory tests.

Patients with suspected pleural TB (any TB symptoms, including cough, fever, night sweats, loss of weight, hemoptysis, and/or chest pain, and features consistent with a pleural effusion on chest X-ray) were prospectively recruited from Groote Schuur Hospital in Cape Town, South Africa. The University of Cape Town Human Research Ethics Committee approved the study (HREC 421/2006 and 919/2014). All patients provided informed consent for study participation.

Pleural fluid was collected by ultrasound-guided pleurocentesis. A closed pleural biopsy, although not routine, was performed using an Abrams needle to aid in patient categorization. Biopsy specimens were collected following aspiration of pleural fluid. Pleural fluid samples were subjected to routine biochemical and cytological analysis by the National Health Laboratory Services (NHLS). This included protein, albumin, ADA, glucose, differential cell counts, cytology, concentrated fluorescence smear microscopy, and liquid culture for M. tuberculosis using the MGIT 960 (Becton, Dickinson, Sparks, MD). Pleural fluid ADA levels of >30 U/liter were reported as suggestive of pleural TB in accordance with national guidelines (18). The remaining fluid was placed in a biobank, frozen at −80°C, and subsequently used for ULTRA, Xpert MTB/RIF, and IRISA-TB analyses. Pleural biopsy samples were sent for histology and/or liquid culture. When possible, sputum was also collected for routine smear microscopy and liquid culture by the NHLS. HIV testing was performed in consenting patients.

Due to the limitations of a single pleural fluid TB culture for confirming a diagnosis, a composite reference standard was used for patient categorization (this reference standard was used in all analyses presented). Patients were categorized as (i) definite TB, i.e., patients with at least one positive M. tuberculosis culture (pleural fluid, biopsy specimen, and/or sputum) and/or caseating granulomatous inflammation suggestive of TB on histological examination of pleural biopsy tissue and with improvement on anti-TB treatment (all patients in this category received anti-TB treatment), (ii) probable TB, i.e., patients not meeting the criteria for definite TB but with clinical and radiological indicators suggestive of TB and who were initiated on and responded to anti-TB treatment (all patients in this category received anti-TB treatment), and (iii) non-TB, i.e., patients with no microbiological or histological evidence of M. tuberculosis and/or for whom an alternative diagnosis was available. These patients did not receive anti-TB treatment either at presentation or on follow-up.

### IFN-γ measurement.

Interferon gamma concentrations were measured in pleural fluid supernatants using the IRISA-TB assay (Antrum Biotech Pty Ltd., Cape Town, South Africa) according to the manufacturer’s instructions. The assay was performed in duplicate and the average values reported. Pleural fluid supernatant was prepared by centrifuging 1 ml of pleural fluid at 3,000 × *g* for 15 min.

### ULTRA and Xpert MTB/RIF assays.

Both the ULTRA and Xpert MTB/RIF assays were performed using 1 ml of pleural fluid diluted with 2 ml of Xpert sample buffer, followed by vigorous mixing. ULTRA and Xpert MTB/RIF cartridges were run on a GeneXpert 4-module machine (Dx System, version 4.7b; Cepheid). To evaluate the effect of sample concentration on ULTRA and Xpert MTB/RIF sensitivity, a median (interquartile range [IQR]) of 10 (5 to 10) ml pleural fluid was centrifuged at 3,000 × *g* for 15 min, and the corresponding pellet was resuspended in 1 ml of PBS. The sample was then processed as described for unconcentrated samples. PCR inhibition was evaluated by comparing the PCR cycle threshold (*C_T_*) values of the internal positive control (lyophilized Bacillus atrophaeus subsp. *globigii* spores) from neat (undiluted) and concentrated samples. The limit of detection (LOD) of ULTRA and Xpert MTB/RIF was determined in triplicate by serially diluting H37Rv CFU (0 to 125 CFU/ml) into 1-ml aliquots of non-TB pleural fluid sample. An H37Rv stock solution was aspirated several times using a fine-gauge needle to prevent aggregation, followed by performing serial dilutions into a 0.25% Tween 80–phosphate-buffered saline (PBS) solution, as performed in previous studies (17, 19, 20). Equal volumes of each dilution were then added to 1 ml of non-TB pleural fluid samples in triplicate. The CFU/ml of each dilution was confirmed by enumeration on oleic acid-albumin-dextrose-catalase (OADC)-enriched 7H10 agar.

### Statistical analysis.

Diagnostic accuracy, including 95% confidence intervals (95% CIs), was assessed using sensitivity, specificity, positive predictive value (PPV), negative predictive value (NPV), and area under the receiver operator characteristic curve (AUROC) in definite TB and non-TB groups. Unpaired and paired categorical variables were compared using the χ^2^ and McNemar test, respectively. Continuous variables were compared using Student's *t* test where appropriate. The Mann-Whitney and Wilcoxon rank sum test was used for unpaired and paired nonparametric continuous variables, respectively. Statistical analyses were performed using GraphPad Prism (version 6.0), Medcalc, version 18.6, and Microsoft Excel. PPV, NPV, and likelihood ratios were compared by DEAD, Semiquant (https://semiquant.shinyapps.io/DEAD/).

## RESULTS

### Clinical and demographic data.

**FIG 1:**
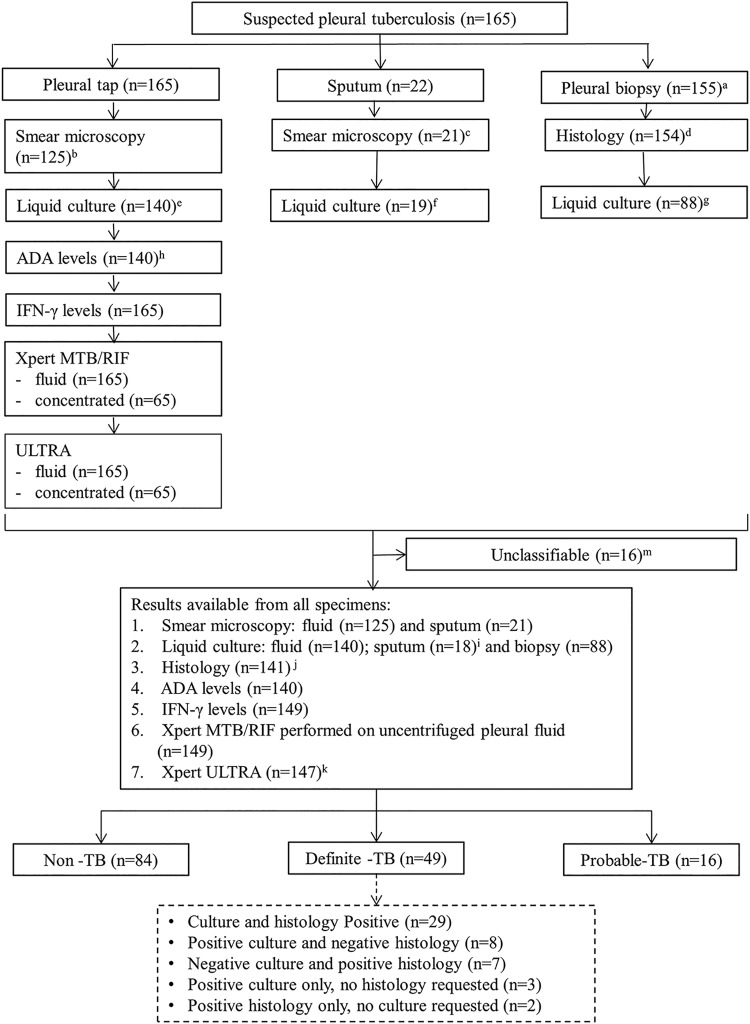
Study overview of patient groups, investigations performed, and tests undertaken. Superscript letters: a, no biopsy specimen taken, *n* = 10; b, fluid smear not requested, *n* = 40; c, sputum smear not requested, *n* = 1; d, histology not requested, *n* = 11; e, fluid culture not requested, *n* = 25; f, sputum culture not requested, *n* = 3; g, biopsy culture not requested, *n* = 66; h, ADA levels not requested, *n* = 25; i, contamination, *n* = 1; j, biopsy sample suboptimal for histology, *n* = 13; k, errors, *n* = 2; m, insufficient clinical data for final diagnosis. Participants classifications: definite TB, at least one positive M. tuberculosis culture (pleural fluid, biopsy specimen, and/or sputum) and/or caseating granulomatous inflammation suggestive of TB on histological examination of pleural biopsy tissue and with improvement on anti-TB treatment; probable TB, patients not meeting the criteria for definite TB but with clinical and radiological indicators suggestive of TB and who were initiated on and responded to anti-TB treatment; non-TB, patients with no microbiological or histological evidence of M. tuberculosis and/or an alternative diagnosis was available.

A total of 165 patients were recruited into the study; 49 subjects had definite TB, 84 were classified as non-TB, and 16 subjects were classified as probable TB. Those with non-TB effusions had a spectrum of malignant and nonmalignant diagnoses, including lymphoma, adenocarcinoma, small-cell carcinoma, and parapneumonic effusion. An additional 16 patients had insufficient clinical data to be categorized in the above-described groups and were subsequently excluded. A study overview is provided in Fig. 1. Demographic and clinical data are summarized in Table 1.

**TABLE 1:** Baseline characteristics of the definite, probable, and non-TB groups

Demographic data	Value(s) for^*a*^:		
	Definite TB (*n* = 49)	Non-TB (*n* = 84)	Probable TB (*n* = 16)
Median age [yr (IQR)]	39a (28–57)	61ab (54–69)	47b (38–53)
Sex [no. (%)]			
Male	32 (21.5%)	54 (36.2%)	10 (6.7%)
Female	17 (11.4%)	30 (20.1%)	6 (4.0%)
HIV infected [no. (%)]			
Yes	9 (6.5%)	4 (2.9%)	4 (2.9%)
No	29 (20.9%)	45 (32.4%)	8 (5.8%)
Unknown	5 (3.6%)	15 (10.8%)	1 (0.7%)
Not tested	5 (3.9%)	13 (9.4%)	1 (0.7%)
Median CD4 count^*b*^ (cells/ml) (IQR)	102^*c*^ (73–232)	117 (39–493)	163 (57–462)
Previous TB [no. (%)]			
Yes	9 (6.0%)	9 (6.0%)	5 (3.4%)
No	32 (21.5%)	61 (40.9%)	7 (4.7%)
Unknown	8 (5.4%)	14 (9.4%)	4 (2.7%)

a Continuous data were analyzed by unpaired *t* test; categorical data were analyzed by chi-square test. Letters a and b were used to indicate which groups were being compared for statistical analysis. *P* < 0.0001.

b CD4 counts are available for all HIV-infected individuals unless otherwise stated.

c One definite HIV-infected TB patient did not have an available CD4 count result. As such, the median CD4 counted is reported for 8 patients. See Table S1 for the number of definite TB participants that were culture and histology positive, culture negative and histology positive, culture positive and histology positive, culture positive with no histology requested, and histology positive with culture requested.

### Performance outcomes of IRISA-TB.

**FIG 2:**
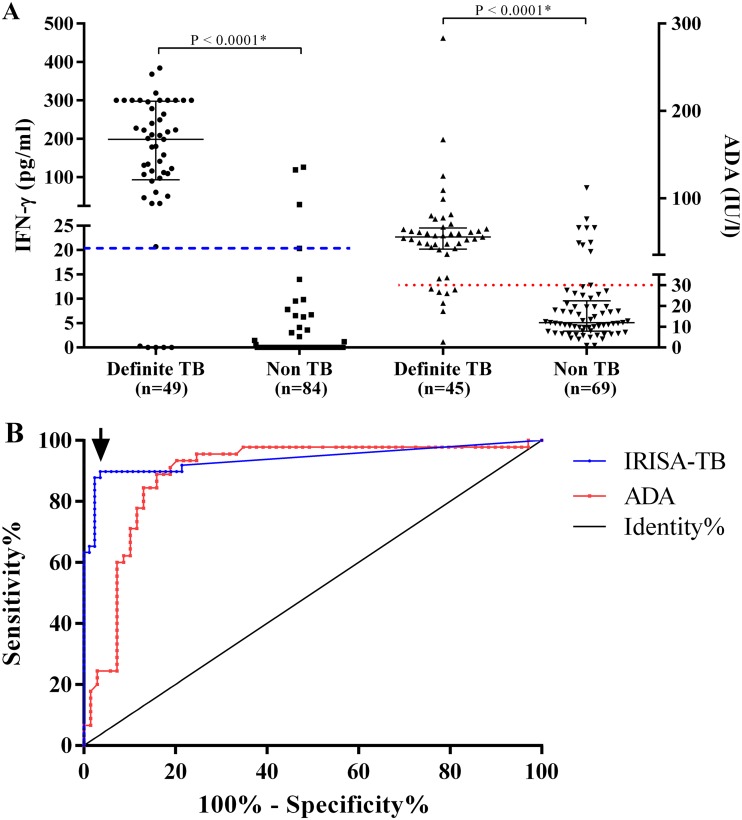
(A) Scatter plot of IFN-γ levels using IRISA-TB and adenosine deaminase (ADA) using pleural fluid from patients with definite TB and non-TB pleural effusions. *, *P* value determined by Mann-Whitney test. Receiver operator characteristic (ROC)-derived cut point of 20.5 pg/ml IFN-γ (indicated by red dotted line) for IRISA-TB and ADA cut point of 30 IU/liter (indicated by blue dashed line). (B) Area under the ROC curves for IRISA-TB and ADA. Areas under the curve were 0.94 (IRISA-TB) and 0.88 (ADA). The ROC curves where generated using the definite TB and non-TB groups, with the chosen cut point for IRISA-TB indicated with an arrow. No significant difference was observed between the two ROC curves by the Hanley and McNeil method.

The median (IQR) IFN-γ levels (*n* = 149) were significantly higher in definite TB than non-TB pleural effusions (198.7 pg/ml [93.4 to 298.2] versus 0.0 pg/ml [0.0 to 0.0], *P* < 0.0001) (Fig. 2A). Using definite and non-TB groups, an ROC curve-derived rule-in cut point of 20.5 pg/ml (the sensitivity, specificity, PPV, and NPV of IRISA-TB [all with 95% CI] were 89.8% [81.3 to 98.3], 96.4% [92.4 to 100], 93.6% [86.6 to 100], and 94.2% [89.2 to 99.1], respectively) (Fig. 2B). Table 2 compares the diagnostic accuracy of IRISA-TB with that of other same-day diagnostics in the definite TB versus non-TB groups.

**TABLE 2:** Accuracy of Xpert G4, ULTRA, IRISA-TB, and ADA for the diagnosis of pleural TB^*a*^

Assay	Values [% (CI), *n*/*N*] for:				Ratio (CI)		
	Sensitivity	Specificity	PPV	NPV	Positive likelihood	Negative likelihood	Diagnostic odds
Xpert ULTRA	37.5^bd^ (23.8–51.2), 18/48	98.8^f^ (96.5–100), 83/84	94.7^j^ (84.7–100), 18/19	73.5^nk^ (65.3–81.6), 83/113	31.5 (4.3–228.6)	0.6^qt^ (0.5–0.8)	49.8 (6.4–389.4)
Xpert MTB/RIF	28.6^ac^ (15.9–41.2), 14/49	98.8^e^ (96.4–100), 83/84	93.3^i^ (80.7–100), 14/15	70.3^ml^ (62.1–78.6), 83/118	24.0 (3.2–177.0)	0.7^sr^ (0.6–0.9)	33.2 (4.2–262.3)
IRISA-TB, cut point of 20.5 pg/ml	89.8^ab^ (81.3–98.3), 44/49	96.4^g^ (92.4–100), 81/84	93.6^h^ (86.6–100), 44/47	94.2^kl^ (89.2–99.1), 81/86	25.1^p^ (8.2–76.7)	0.1^qs^ (0.0–0.2)	237.6 (54.2–1041.3)
ADA, cut point of 30 IU/ml	84.4^cd^ (73.9–95.0), 38/45	87.5^feg^ (79.9–95.1), 63/72	80.9^hij^ (69.6–92.1), 38/47	90.0^mn^ (83.0–97.0), 63/70	6.8^p^ (3.6–12.6)	0.2^rt^ (0.1–0.4)	38.0 (13.1–110.4)

a Positive M. tuberculosis pleural fluid, biopsy specimen, and/or sputum culture and/or histology in keeping with M. tuberculosis infection was used as a reference for definite TB. No microbiological or histological evidence of M. tuberculosis and/or an alternative diagnosis being available was defined as non-TB. IRISA-TB IFN-γ cut point of 20.5 pg/ml and ADA clinical cut point of 30 IU/liter were used for clinical decision-making. Letters a, b, c, d, e, f, g, h, I, j, k, l, m, n, p, q, r, s, and t were used to indicate which groups were being compared for statistical analysis. a, b, c, and d, *P* < 0.0001; e, *P* = 0.004; f, *P* = 0.005; g, *P* = 0.034; h, *P* = 0.028; i, *P* = 0.071; j, *P* = 0.032; k, l, and m, *P* < 0.0001; n, *P* = 0.00013; p, *P* = 0.032; q, r, and s, *P* < 0.0001; t, *P* = 0.0006.

### Performance outcomes of pleural fluid ADA.

Median (IQR) ADA levels (*n* = 140) were approximately 5 times higher in definite TB than non-TB effusions (55.6 [41.7 to 65.9] versus 12.0 [1.0 to 22.4] U/liter; *P* < 0.0001). Using a clinical cut point of >30 U/liter (18), the sensitivity, specificity, PPV, and NPV (all with 95% CI) of ADA were 84.4% (73.9 to 95.0), 87.5% (79.9 to 95.1), 80.9% (69.6 to 92.1), and 90.0% (83.0 to 97.0), respectively (Table 2). The scatter plot and ROC of ADA are shown in Fig. 2A and B.

### Performance outcome of ULTRA and Xpert MTB/RIF.

The sensitivity (95% CI) of ULTRA (*n* = 149) was marginally better than that of Xpert MTB/RIF (37.5% [23.8 to 51.2] versus 28.6% [15.9 to 41.2], *P* = 0.393) (Table 2). Pleural fluid concentration did not significantly improve the sensitivity of either ULTRA or Xpert MTB/RIF (29.5% versus 31.3% and 29.5% versus 33.4%) (Table 3). The median (IQR) *C_T_* values of the Xpert MTB/RIF internal positive control were significantly different between neat and concentrated samples (26.3 versus 25.55, *P* = 0.0483) but not when using ULTRA (see Fig. S1 in the supplemental material). ULTRA had a lower LOD than Xpert MTB/RIF (18.7 CFU/ml versus ≥76.2 CFU/ml of pleural fluid, respectively) (Fig. S2). Furthermore, the lowest dilution to provide a trace-positive result by ULTRA was 8.8 CFU/ml.

**TABLE 3:** Sensitivity and specificity of ULTRA and Xpert MTB/RIF assay using unprocessed and concentrated (pellet centrifugation) pleural fluid to diagnose pleural TB^*a*^

Sample type	Values [% (CI), *n*/*N*] for:			
	ULTRA		Xpert MTB/RIF	
	Sensitivity	Specificity	Sensitivity	Specificity
Fluid	29.5 (13.3–53.2), 5/17	100 (89.6–100), 34/34	29.5 (13.3–53.2), 5/17	100 (89.9–100), 34/34
Concentrated	31.3 (14.2–55.6), 5/16^*b*^	100 (89.6–100), 33/33	33.4 (15.2–58.3), 5/15^*c*^	100 (89.3–100), 32/32

a Two aliquots of a median volume of 10 ml of pleural fluid was centrifuged at 3,000 × g for 15 min with the pellet resuspended in sterile PBS, followed by Xpert MTB/RIF and ULTRA. A positive M. tuberculosis pleural fluid, biopsy specimen, and/or sputum culture and/or histology in keeping with M. tuberculosis infection was used as a reference for definite TB. No microbiological or histological evidence of M. tuberculosis and/or an alternative diagnosis being available was defined as non-TB.

b Error (*n* = 1) in the concentrated ULTRA.

c Error (*n* = 2) in the concentrated Xpert MTB/RIF.

## DISCUSSION

Given that a reliable same-day diagnostic tool for pleural TB is still lacking, we prospectively evaluated the utility of ADA, IRISA-TB, Xpert MTB/RIF, and the recently released ULTRA assay for the diagnosis of pleural TB. Our key findings were that (i) ULTRA sensitivity was no better than that of the conventional Xpert MTB/RIF despite a lower *in vitro* limit of detection, (ii) the ULTRA sensitivity was not improved by pelleting of larger volumes of pleural fluid, (iii) ADA and IRISA-TB had significantly higher sensitivity for pleural TB than molecular tests, and (iv) compared to those of ADA, IRISA-TB had significantly better specificity and positive predictive value, making it the ideal rule-in test for pleural TB (although it also had a very high NPV in a high-burden setting, prompting clinicians to search for alternative diagnoses that may mandate pleural biopsy and thoracoscopy). There are a number of strengths and novel aspects of our study. It is the first study to comprehensively evaluate Xpert Ultra in patients with suspected pleural TB (and directly against the Xpert G4 cartridge), it is the largest study to date (149 participants) to evaluate a nucleic acid amplification test, ADA, and unstimulated IFN-γ in tandem, it is the first study to evaluate ULTRA for pleural TB in the context of HIV coinfection, and it evaluated an updated version of the IFN-γ assay. The use of a composite reference standard (culture and histopathology) better reflects the true performance of each assay (as culture alone is an imperfect gold standard in this context).

The ULTRA cartridge, which incorporates a larger input sample volume and two different multicopy amplification targets (IS*6110* and IS*1081*), results in an approximately 10-fold improvement in the lower limit of detection *in vitro* using spiked M. tuberculosis (13), yet the sensitivity in clinical samples remained suboptimal and not much different from that of the conventional MTB/RIF cartridge. This is most likely due to the immune-mediated and paucibacilliary nature of pleural TB, which remains below the detection limit (even) of ULTRA. The only published data on ULTRA in pleural TB comes from a low-burden setting using samples from different extrapulmonary sites, and that only included a small number (*n* = 24) of pleural fluid samples (14). Furthermore, culture positivity was used as the reference standard, resulting in sample and selection bias (only 40% of pleural TB is culture positive), which may have overestimated test specificity. Given that culture positivity self-selects for higher burden of microbiological disease, restricting analysis to this subgroup is also likely to overestimate sensitivity. We have also confirmed that the limit of detection of the ULTRA was 10-fold lower than that of Xpert MTB/RIF (8.8 versus 76.2 CFU/ml, respectively). Pleural fluid is known to have inhibitory molecules which can affect molecular assays (21). However, no PCR inhibition of the positive internal control was seen when using the ULTRA assay, whereas inhibition was seen with the Xpert MTB/RIF assay (but not in a previous study that we performed, presumably due to a sample size effect) (17). Pellet-based concentration of the pleural fluid by centrifugation and resuspension did not improve sensitivity of either ULTRA or Xpert MTB/RIF. The median time to positivity was 22 days, indicating a low bacterial load within the fluid. Furthermore, ULTRA-positive, culture-positive samples tended to have a shorter time to positivity than the ULTRA-negative, culture-positive ones (data not shown), confirming the perception that pleural fluid is highly paucibacillary (and concentrating 10 ml of pleural fluid is unlikely to improve performance despite ULTRA being more sensitive). Moreover, concentrating volumes larger than 10 ml is unlikely to improve sensitivity, as centrifuging as much as 100 ml of fluid does not improve the diagnostic yield of culture (22), which has a limit of detection similar to that of ULTRA (13, 23). This is in contrast to TB meningitis and genitourinary/disseminated TB in advanced HIV, where concentrating the cerebrospinal fluid (CSF) or urine fluid improves the sensitivity of Xpert MTB/RIF (24, 25). This is likely because TB serositis is more of an immune-reactive disease characterized by a hypersensitivity reaction to TB antigens in addition to mycobacterial invasion of the pleural space. Thus, while we have previously shown that CSF (24) and urine (25) centrifugation may improve sensitivity, concentration in the case of a hypersensitivity reaction will have little effect given the very low burden of mycobacteria or TB antigen. The same phenomenon likely explains the lack of a concentration effect in TB pericarditis, as we have previously shown (26).

In high-TB-burden settings such as South Africa, a high ADA level (cut point of 30 IU/liter) is frequently used to guide initiation of anti-TB treatment. In this study, using the accepted laboratory cut point of 30 IU/liter, about one-fifth of the TB patients would have been missed, and close to 1 in every 10 non-TB patients would have erroneously been initiated on unnecessary anti-TB treatment. In high-prevalence settings, ADA has satisfactory diagnostic performance, but in lower-prevalence settings the PPV is not clinically useful (27). The most recent meta-analysis reported sensitivity and specificity of 86% and 88%, respectively (28), confirming the misclassification bias and that 1 out of every 9 or 10 non-TB patients would be erroneously placed on anti-TB treatment (at a 10% disease prevalence this would amount to about 10 additional false TB starts in every 100 patients suspected with pleural TB). The specificity of ADA can be improved if the proportion of lymphocytes is taken into account (27). However, this was not routinely requested by the attending clinician and was not expressly part of our study protocol. Furthermore, a significant proportion (∼25%) of pleural effusions is neutrophil predominant (29), and lymphocyte counts in pleural fluid are not widely accessible (for the same reason it is not frequently requested in our setting). As such, this analysis was not performed.

IRISA-TB sensitivity, like that of ADA, was significantly higher than that of ULTRA, highlighting that tests requiring the express detection of M. tuberculosis will always struggle to reach the sensitivity of immunodiagnostic tests (which can greatly reduce the need for invasive biopsy procedures). Indeed, we have confirmed our previous findings, and those of others, that IFN-γ is both a good rule-in and rule-out diagnostic test for pleural TB (16, 17, 30–32). We used the IRISA-TB kit, which is both rapid and inexpensive and can be used in most resource-poor settings where other routine enzyme-linked immunosorbent assays are performed; moreover, it gets around the hurdle of long assay times and high cost of research-based kits, which remain unvalidated in a clinical setting. The latter is important, as EPTB compartments have high concentrations of interfering heterophile molecules and, thus, kit-based variation in sensitivity can be considerable (33–35). Interferon gamma levels were also found to be elevated in three non-TB patients. Two of these three patients showed similar histopathology and ADA levels, but there was no alternative clinical diagnosis to explain the IFN-γ results with the available clinical information. One drawback of using immunodiagnostic tests is the lack of antimicrobial susceptibility data, which requires either a culture isolate or positive nucleic acid amplification test. However, the diagnostic yield in pleural fluid for both is low, making this concern redundant. The diagnostic yield can be improved with pleural biopsy specimens (30). Indeed, in the current study pleural fluid culture sensitivity (45%) was lower than biopsy culture sensitivity (82%; data not shown). Recently, Christopher et al. showed a 30% increase in Xpert MTB/RIF sensitivity when using pleural tissue in addition to pleural fluid, i.e., macerated pleural tissue was used in the Xpert assay (36). This approach was not undertaken in our study but still would have meant that ULTRA sensitivity was in the region of ∼50%. Further studies are required to interrogate this issue, although its importance is mitigated by the fact that pleural biopsy is not routinely performed in most settings where TB is endemic.

There are several limitations to our study. There was a low proportion of HIV-infected patients, and many patients had unknown HIV status. However, the HIV prevalence rates among TB patients in the Western Cape Province are known to be lower than those in the rest of South Africa (37), and patients often refuse testing. Nevertheless, our findings were still derived in a setting where TB is endemic with a relatively high HIV coinfection rate where Beijing strains predominate, which ideally would be confirmed in other settings. A further limitation is that we did not evaluate the potential impact on morbidity and length of hospital stay of ADA, IRISA-TB, Xpert MTB/RIF, and ULTRA compared to those of empirical treatment. However, our study design did not lend itself to deriving these measures (it would have required a randomized controlled trial), and an interventional study design would have been difficult to interpret because of high rates of empirical treatment. Lastly, as TB-IRISA was performed on frozen pleural fluid samples, it is possible that cell lysis due to freeze/thaw resulted in slightly inflated IFN-γ levels compared to those of freshly run samples. However, we believe this effect is negligible based on correspondence with the manufacturer and because IFN-γ protein is rapidly released from cells (other methods of IFN-γ detection, such as flow cytometry, require protocols that inhibit the secretion of intracellular cytokines to reliably detect them). The effect of freeze/thaw on ADA is also uncertain.

In conclusion, despite a better limit of detection than the conventional Xpert MTB/RIF cartridge, ULTRA has poor sensitivity for the diagnosis of pleural TB. Biomarkers such as ADA and IRISA-TB are significantly more sensitive, with IRISA-TB demonstrating a higher specificity and rule-in value than ADA in a setting where TB and HIV are endemic.

## Supplementary Material

Supplemental file 1
