# Supplementary material for: Same-Day Tools, Including Xpert Ultra and IRISA-TB, for Rapid Diagnosis of Pleural Tuberculosis: a Prospective Observational Study
Source: J Clin Microbiol. 2019 Aug 26;57(9):e00614-19. doi: 10.1128/JCM.00614-19 (PMC6711909; doi:10.1128/JCM.00614-19)
Supplement: Supplemental file 1 [file JCM.00614-19-s0001.pdf]

ONLINE DATA SUPPLEMENT

Same day tools, including Xpert Ultra and unstimulated IFN-γ, for the rapid diagnosis of pleural tuberculosis – a prospective observational study.

Richard Meldau[1], Philippa Randall[1], Anil Pooran[1], Jason Limberis[1], Edson Makambwa[1], Muhammed Dhansay[1], Ali Esmail[1] and Keertan Dheda[12#]

[1] Centre for Lung Infection and Immunity, Division of Pulmonology, Department of Medicine and UCT Lung Institute, University of Cape Town, Cape Town.

[2] London School of Hygiene and Tropical Medicine, London, United Kingdom.

[#]Corresponding author: Keertan Dheda

Postal Address: Centre for Lung Infection and Immunity, Department of Medicine & UCT Lung Institute, University of Cape Town, South Africa. H Floor, Room H46.41 Old Main Building, Groote Schuur Hospital, Groote Schuur Drive, Observatory 7925

E-mail: Keertan.dheda@uct.ac.za

Tel: +27 21 404 7654

Fax: +27 21 650 3824

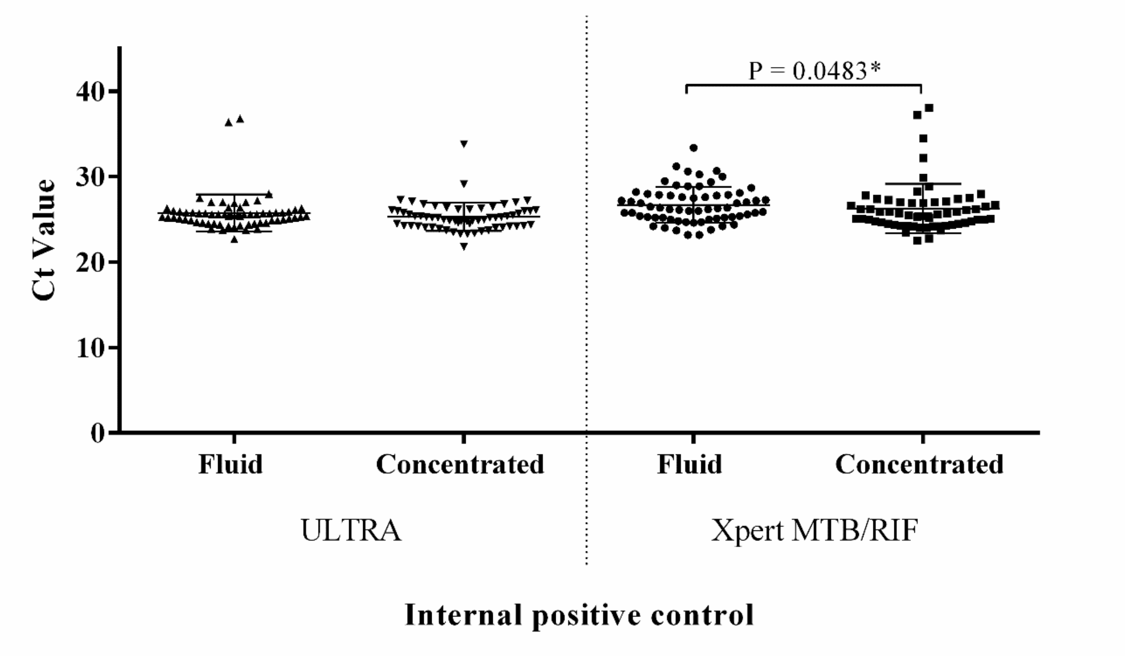

Figure S1: The effect of concentration (pellet centrifugation) on the PCR cycle-threshold (CT) values of the internal positive control of ULTRA and Xpert G4. * Wilcoxon pair-matched.

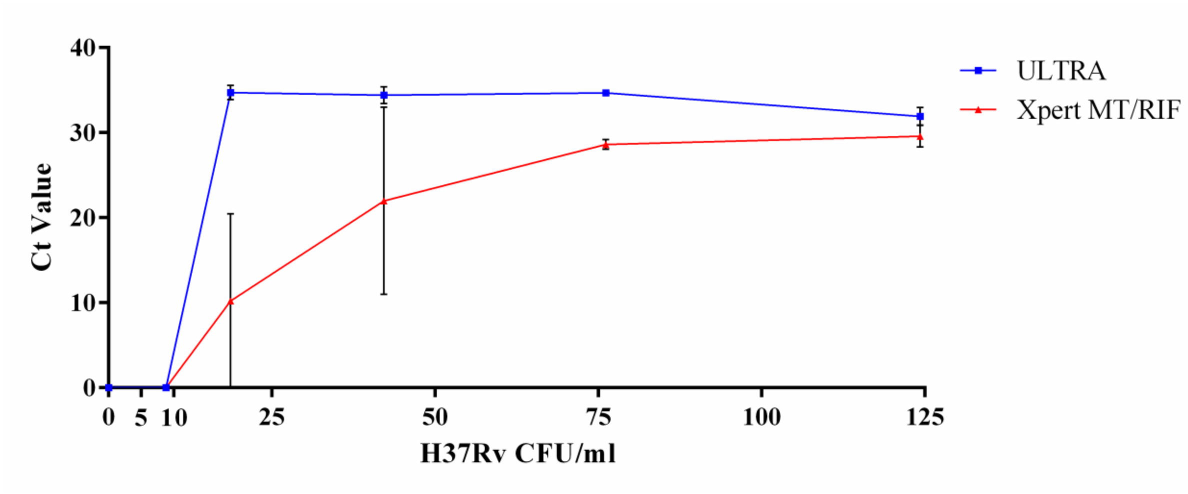

Figure S2: The determination of the limit of detection of ULTRA and conventional Xpert MTB/RIF cartridges in pleural fluid. Each assay was performed in triplicate for each dilution.

Table S1: Xpert ULTRA for the diagnosis pleural tuberculosis stratified according to HIV status

	**Sensitivity % (CI) n/N**	**Specificity % (CI) n/N**	**Positive Predictive Value % (CI) n/N**	**Negative Predictive Value % (CI) n/N**	**Positive Likelihood Ratio (CI)**	**Negative Likelihood Ratio (CI)**	**Diagnostic odds Ratio (CI)**
**HIV uninfected**	32.2 (18 – 50.7) 9/28	97.8 (88.5 - 99.7) 44/45	90.0 (59.6 – 98.3) 9/10	69.9 (57.7 – 79.8) 44/63	14.9 (2.0– 110.6)	0.7 (0.5 – 0.9)	21.3 (2.5 – 180.2)
**HIV infected**	55.6 (26.7 – 81.2) 5/9	100 (51.1 - 100) 4/4	100 (56.6 - 100) 5/5	50 (21.6 – 78.5) 4/8	1.11 (0.4 – 2.8)	0.4 (0.2 – 0.9)	1.3 (0.2 – 8.4)

A positive *M.tb* pleural fluid, biopsy and/or sputum culture and/or histology in keeping with *M.tb* infection used as a reference for Definite TB. No microbiological or histological evidence of *M.tb* and/or an alternative diagnosis was available was defined as Non-TB. CI: confidence interval.

Table S2: IRISA-TB for the diagnosis pleural tuberculosis stratified according to HIV status

	Sensitivity % (CI) n/N	Specificity % (CI) n/N	Positive Predictive Value % (CI) n/N	Negative Predictive Value % (CI) n/N	Positive Likelihood Ratio (CI)	Negative Likelihood Ratio (CI)	Diagnostic odds Ratio (CI)
**HIV uninfected**	89.7<br>(73.7 – 96.5)<br>26/29	95.6<br>(85.2 – 98.8)<br>43/45	92.9<br>(77.4 – 98.1)<br>26/28	93.5<br>(82.5 – 97.8)<br>43/46	20.2<br>(5.2 – 78.6)	0.1<br>(0 – 0.3)	186.3<br>(29.2 – 1190.1)
**HIV infected**	88.9<br>(56.6 - 98.1)<br>8/9	100<br>(51.1 - 100)<br>4/4	100<br>(67.6 - 100)<br>8/8	80<br>(37.6 – 96.4)<br>4/5	Incalculable	0.1<br>(0 – 0.7)	Incalculable

A positive *M.tb* pleural fluid, biopsy and/or sputum culture and/or histology in keeping with *M.tb* infection used as a reference for Definite TB. No microbiological or histological evidence of *M.tb* and/or an alternative diagnosis was available was defined as Non-TB. CI: confidence interval.

Table S3: ADA for the diagnosis pleural tuberculosis, stratified according to HIV status

	**Sensitivity % (CI) n/N**	**Specificity % (CI) n/N**	**Positive Predictive Value % (CI) n/N**	**Negative Predictive Value % (CI) n/N**	**Positive Likelihood Ratio (CI)**	**Negative Likelihood Ratio (CI)**	**Diagnostic odds Ratio (CI)**
**HIV uninfected**	76 (56.6 – 88.6) 19/25	92.4 (79.7 – 97.4) 36/39	86.4 (66.7 – 95.3) 19/22	85.8 (72.2 - 93.3) 36/42	9.9 (3.3 – 30)	0.3 (0.1 – 0.5)	38 (8.5 – 169.1)
**HIV infected**	100 (70.1 - 100) 9/9	100 (43.9 - 100) 3/3	100 (70.1 - 100) 9/9	100 (43.9 - 100) 3/3	Incalculable	0	Incalculable

A positive *M.tb* pleural fluid, biopsy and/or sputum culture and/or histology in keeping with *M.tb* infection used as a reference for Definite TB. No microbiological or histological evidence of *M.tb* and/or an alternative diagnosis was available was defined as Non-TB. CI: confidence interval.
